# Prostate Cancer Screening in Brazil: a single center experience in the public health system

**DOI:** 10.1590/S1677-5538.IBJU.2020.0392

**Published:** 2020-12-20

**Authors:** Renato Almeida Rosa de Oliveira, Gustavo Cardoso Guimarães, Thiago Camelo Mourão, Ricardo de Lima Favaretto, Thiago Borges Marques Santana, Ademar Lopes, Stenio de Cassio Zequi

**Affiliations:** 1 Hospital Beneficência Portuguesa de São Paulo Departamento de Uro-Oncologia São PauloSP Brasil Departamento de Uro-Oncologia, Hospital Beneficência Portuguesa de São Paulo, São Paulo, SP, Brasil; 2 AC Camargo Cancer Center Divisão de Urologia São PauloSP Brasil Divisão de Urologia, AC Camargo Cancer Center, São Paulo, SP, Brasil; 3 Hospital Beneficência Portuguesa de São Paulo São PauloSP Brasil Serviço de Oncologia Cirúrgica, Hospital Beneficência Portuguesa de São Paulo, São Paulo, SP, Brasil; 4 AC Camargo Cancer Center São PauloSP Brasil Serviço de Cirurgia Pélvica do AC Camargo Cancer Center, São Paulo, SP, Brasil

**Keywords:** Prostate cance, familial [Supplementary Concept], Mass Screening, Therapeutics

## Abstract

**Purpose:**

Incidence and mortality of prostate cancer (PCa) are still increasing in developing countries. Limited access to the health system or more aggressive disease are potential reasons for this. Ethnic and social differences in developed countries seem to make inappropriate to extrapolate data from other centers. We aim to report the epidemiological profile of a PSA-screened population from a cancer center in Brazil.

**Materials and Methods:**

We retrospectively selected 9.692 men enrolled in a PCa prevention program, comprising total PSA level and digital rectal examination at the first appointment, associated with complementary tests when necessary. Men aged over 40 years-old were included after shared decision-making process. Prostate biopsy (TRUS) was performed when clinically suspected for PCa. After the diagnosis, patients underwent appropriate treatment.

**Results:**

TRUS was performed in 5.5% of men and PCa incidence was 2.6%. Overall ratio between number of patients who needed to be screened in order to diagnose one cancer was 38.9 patients, with 2.1 biopsies performed to diagnose a cancer. Positive predictive value (PPV) of TRUS biopsy in this strategy was 47.2%, varying from 38.5% (<50 years-old) to 60% (>80 years-old). We evidenced 70 patients (27.9%) classified as low risk tumors, 74 (29.5%) as intermediate risk, and 107 (42.6%) as high-risk disease.

**Conclusions:**

PSA-screening remains controversial in literature. In front of a huge miscegenated people and considering the big proportion of high-risk PCa, even in young men diagnosed with the disease, it is imperative to inform patients and health providers about these data particularities in Brazil.

## INTRODUCTION

Prostate cancer (PCa) is the second most common malignancy among men, with an incidence of more than 1.200.000 cases per year worldwide. About seventy percent of the cases are diagnosed in the most developed countries (1). In Brazil, more than 65.000 new cases are estimated in 2020, corresponding to the second most lethal neoplasia after lung cancer (2). Although the treatment methods have been improved (3) and the use of PSA-screening strategies had shown decreasing in mortality rates in developed countries (4), several European and North American studies such as PLCO (5), the Cochrane meta-analysis (6), the ERSPC (7),^8^), and the Göteborg trial (Sweden) (9), the studies of the PIVOT trial (10),^11^), and the UK ProtecT (12),^13^) showed conflicting data.

Ethnic and social differences among populations can confer different results regarding PCa presentation and behavior (14),^15^). Incidence and mortality rates are still increasing in developing countries (16), which may be related to a more aggressive disease or to the limited access to public or private health care systems (15), making data extrapolation from more developed countries potentially inappropriate (17). This can be showed by previously published studies in the Brazilian population, showing higher incidences of locally advanced and metastatic PCa (18),^19^) compared to the North American people (20).

This study aims to describe the PCa epidemiological profile and results of a screening program in a single cancer center regarding a PSA-screened male population from the city of São Paulo, whose population is a representative portion of Brazil, due to its geographical and economic characteristics determined by migratory and immigration issues. Moreover, we make a discussion related to the results of some international PCa screening trials.

## MATERIALS AND METHODS

We retrospectively reviewed the registries of 12.969 men spontaneously enrolled in a cancer prevention program in 2013 at a Brazilian reference cancer center. For PCa screening, all men were included after a shared decision-making process. In this study, we selected only men aged over 40 years old with no upper age limit. We excluded patients with a prior cancer diagnosis. No other exclusion criteria were considered. Thus, we selected for analysis 9.692 men who underwent a PSA-screening strategy comprising a total blood serum PSA test and a digital rectal examination (DRE) at the first appointment performed by the medical (non-urological) staff. Complementary tests were associated when deemed necessary.

**Figure 1:**
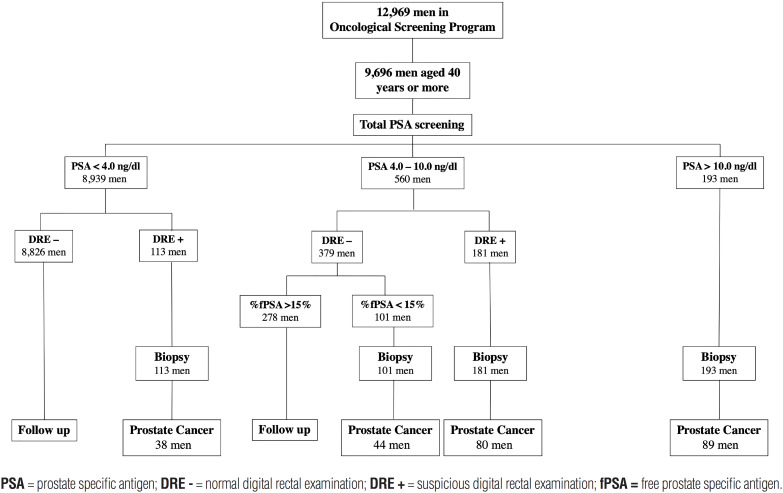
PSA-screening program flowchart.

Patients with a total PSA level greater than 10.0ng/dL or a suspected DRE for the presence of malignancy were referred to a transrectal ultrasound-guided (TRUS) prostate biopsy. Patients with a total PSA level between 4.0 and 10.0ng/dL and a normal DRE underwent a serum free-PSA level test (fPSA). In these cases, TRUS was performed when the percentage ratio (fPSA/total PSA level) was below 15%. Patients with a total PSA level below 4.0ng/dL and a normal DRE were discharged to primary care follow-up. Considering that patients with a total PSA >10.0ng/dL would be referred to TRUS biopsy, DRE was not performed in these men by the non-urological staff. Figure-1 describes the program flowchart.

All laboratory and image tests, TRUS biopsy and therapeutic procedures were performed at the same institution, exclusively provided by public health system resources. TRUS biopsy protocol consisted of a 12-core biopsy comprising all prostate areas, besides additional samples from suspicious areas on DRE. All cancer specimens were analyzed according to the modified Gleason score (21). All diagnosed patients were submitted to the AJCC - TNM 8th edition staging system (22) and classified according to the D’Amico risk groups. Diagnosed patients were referred to the Department of Urology and staged with multiparametric magnetic resonance imaging (mpMRI) of the prostate and bone scan, according to the discretion of each surgeon. Institutional Review Board approval was obtained for this study (number 2509/18).

### Statistical Analysis

For statistical analysis, we described the distribution of screened and diagnosed men across this period. Continuous variables were stratified in categories (e.g.: age, PSA level). Mean, median, and ranges are reported for descriptive statistics in continuous variables. Categorical variables were expressed as absolute numbers and proportions. Positive predictive value (PPV) for recommended biopsy was assessed by the proportion of diagnosed PCa cases and patients who had the criteria for TRUS biopsy. The ratio of diagnosed cases was defined as the number of patients who needed to be screened in order to diagnose one cancer. All data were analyzed with IBM SPSS Statistics 23.0 (IBM, Inc., Armonk, NY).

## RESULTS

Among the 9.692 men, 532 (5.5%) patients underwent TRUS prostate biopsy, diagnosing 251 cancer cases. PCa overall incidence was 2.6% and positive predictive value (PPV) of TRUS biopsy in this screening strategy was 47.2%. The mean age of PSA-screened patients was 54 years (range=40-89 years old). In this series, 6.274 (64.7%) men were considered white, 2.246 (23.2%) were brown, 610 (6.3%) were black, and 561 (5.8%) were considered yellow (Eastern Asian). Median PSA level was 0.774ng/dL (range=0.1-5.000.00ng/dL). PSA level was below 4.0ng/dL in 92.2% of the cases. Overall ratio between the number of patients who needed to be screened in order to diagnose one cancer was 38.9 patients, with 2.1 biopsies performed to diagnose a cancer. Digital rectal examination was considered suspicious (DRE +) in 294 men with PSA<10.0ng/dL (PPV=40.1%; 118 positive biopsies/294 DRE +). DRE was not performed by the non-urological staff in patients with PSA>10.0ng/dL (193 cases) and therefore 137 patients were referred directly to TRUS biopsy. Fifty-six patients with PSA above 10.0ng/dL did not have clinical conditions to undergo biopsy and they were referred to clinical stabilization before further oncological investigation. Characteristics of the screened men categorized by age groups and PSA level are described in Table-1.

**Table 1:** Distribution of the screened patients stratified according to the age groups and PSA levels.

Total = 9692	40 – 49 years n = 3280 (33.8%)	50 – 59 years n = 3061 (31.6%)	60 – 69 years n = 2284 (23.6%)	70 – 79 years n = 892 (9.2%)	≥ 80 years n = 175 (1.8%)	TRUS prostate biopsy (%)	PCa diagnosed cases	PPV	Ratio
Total PSA level - median (IQR)	0.571 (0.1 – 0.95)	0.76 (0.367 – 1.37)	1.10 (0.50 – 2.36)	1.59 (0.518 – 3.72)	1.82 (0.61 – 4.57)				
PSA level groups (%)									
< 4.0 ng/dL	3236 (98.6)	2922 (95.5)	1969 (86.2)	689 (77.2)	123 (70.3)	113 (1.2)	38	33.6%	235 / 2.9 / 1
4.0 – 10.0 ng/dL	41 (1.3)	111 (3.6)	235 (10.3)	143 (16.1)	31 (17.7)	282 (50.3)	124	44.0%	4.5 / 2.2 / 1
> 10 ng/dL	3 (0.1)	28 (0.9)	80 (3.5)	60 (6.7)	21 (12.0)	137 (71.0)	89	65.0%	2.2 / 1.52 / 1
TRUS prostate biopsy (%)	26 (0.8)	112 (3.7)	243 (10.6)	126 (14.1)	25 (14.3)				
PCa diagnosed cases	10	47	107	72	15				
PPV	38.5%	41.9%	44.1%	57.6%	60%				
Ratio (N / biopsies / 1 cancer)	328 / 2.6 / 1	65.1 / 2.4 / 1	21.3 / 2.3 / 1	7.1 / 1.7 / 1	11.6 / 1.6 / 1				

**IQR** = interquartile range; **PSA** = prostate specific antigen; **TRUS** = transrectal ultrasound-guided; **PPV** = positive predictive value; **PCa** = prostate cancer; **N** = number of screened patients divided by the diagnosed cases

According to the pathological stage, 218 cases (87%) were classified as localized diseases, and only 11 (4.3%) patients were diagnosed with metastatic disease. Median PSA in the PCa group was 7.4ng/dL. Forty-one patients (16.3%) had clinically significant tumors according to the Epstein's criteria (25). The majority of the diagnosed cases were classified as high-risk diseases according to the D’Amico risk classification, as seen in Table-2. Incidence of high-risk tumors varies greatly among the ethnic groups, with 37.3% evidenced in white men, 42.1% in brown men, and 63.3% in black men. The highest incidence of high-risk tumors was evidenced in yellow (Eastern Asians) patients with 72.7% cases.

**Table 2:** Distribution of the diagnosed men stratified according to the D’Amico risk groups.

Total = 251		Low risk n = 70 (27.9%)	Intermediate risk n = 74 (29.5)	High risk n = 107 (42.6%)
**Age groups (%)**				
	40 – 49 years	3 (30%)	2 (20)	5 (50)
	50 – 59 years	21 (44.7)	12 (25.5)	14 (29.8)
	60 – 69 years	25 (23.4)	41 (38.3)	41 (38.3)
	70 – 79 years	17 (23.6)	17 (23.6)	38 (52.8)
	≥ 80 years	4 (26.7)	2 (13.3)	9 (60)
**Ethnic group (%)**				
	White	46 (32.4)	43 (30.3)	53 (37.3)
	Brown	18 (23.7)	26 (34.2)	32 (42.1)
	Black	3 (13.6)	5 (22.7)	14 (63.3)
	Yellow (East Asian)	3 (37.3)	0	8 (72.7)
**Total PSA level - median (IQR) Gleason score groups (%)**		5.48 (4.17 – 7.0)	7.80 (4.31 – 11.59)	12.1 (6.16 – 42.49)
	6	70 (100)	15 (20.3)	2 (1.9)
	7	–	59 (79.7)	46 (43)
	8 - 10	–	–	59 (55.1)
**T stage (%)**				
	Tx	9 (12.8)	2 (2.7)	11 (10.3)
	T1	61 (87.2)	70 (94.6)	77 (72)
	T2	–	2 (2.7)	8 (7.5)
	T3	–	–	7 (6.5)
	T4	–	–	4 (3.7)

**PSA** = prostate-specific antigen; **IQR** = Interquartile range

After referred to the Department of Urology, 205 men underwent a primary treatment at the institution. Forty-six (18.3%) patients subsequently lost to follow-up the appointments. Regarding the patients treated at the institution, 93 (45.4%) men underwent retropubic radical prostatectomy, 23 (11.2%) performed radiation therapy, 45 (22%) performed radiation and hormone therapy, 15 (7.3%) performed exclusive androgen deprivation therapy, 27 (13.2%) men were considered for active surveillance, and 2 (0.9%) were managed with watchful waiting. In the period from 2013 - 2018, five (2.4%) metastatic patients that were submitted to exclusive hormone therapy died. Three deaths were related to the cancer and two had other clinical causes. Oncological and functional outcomes of those treated cases were not the focus of this study.

## DISCUSSION

Benefits of PSA-screening and its real impact on mortality are still controversial, especially after the publication of large studies with conflicting results (5)-13) and the United States Preventive Services Task Force (USPSTF) recommendations (23). The impact on reducing mortality was demonstrated by the European study (7),^8^), with a decrease in the order of 21%, while the PLCO study (5) and the USPSTF (23) did not demonstrate such benefit. Despite allowing early diagnosis of high-risk cases, screening also diagnosis a large number of indolent tumors that potentially do not require immediate intervention, besides to impact in the quality of life and in costs for the health system (24)-26).

Lack of specific and reliable data from developing countries is an aggravating factor for the correct evaluation and strategic planning in the PCa screening in our setting. Intending to a more accurate planning, it is important to have in mind the ethnical distribution and that about 75% of the Brazilian people need the public health system. National governmental agencies in Brazil do not recommend the populational screening. Divergently, Brazilian Society of Urology (SBU) recommends PCa screening in men over 50 years or above 45 years if high risk factors are present (27). Racial distribution shown in our study is similar to the demographic racial data published by Brazilian agency IBGE in “self-denominated skin color” question (28).

PCa incidence in the PSA-screened population was 2.6%, evidencing a higher incidence in relation to the PLCO trial (1.4%) (5), and equivalent to the USPSTF study (2.4%) (23). Our study evidenced an inferior incidence when compared to the Brazilian study from Faria et al. with 3.3% of diagnosed cases (29), and to the ERSPC trial (4.2%) (7),^8^), if considered only the first year of its application. The cumulative incidence of diagnosed cases in the USPSTF, PLCO, ERSPC, and the Göteborg trial (9) was 4.9%, 11%, 10.2%, and 11.5% in 4 years, 13 years, 16 years, and 18 years, respectively.

Positive predictive value (PPV) of TRUS biopsy in this screening strategy was 47.2%, varying from 33.6% to 65% according to the PSA ranges. For PSA >4.0ng/dL, PPV was 50.8%. It was higher than data found in literature, varying from 27.7% to 32% (30),^31^). If considered only cases with suspicious DRE, PPV was 40.1% for patients with PSA <10.0ng/dL. It was also higher than previous publications varying from 17.7% to 21% (30),^31^).

Another enhanced evidence was the high incidence of high-risk cases, which involved 42.6% (107 cases) of PCa patients, also higher than the 23.7% published by Cooperberg et al. in the North American population (17). In addition, we found a high incidence of high-risk tumors in Afro-descendant patients (42.1% - 63.3%), in accordance to the international literature that reports more severe cases among people in this ethnic group (14, 15, 32). On the other hand, Eastern Asians descendants had a 72.7% incidence of high-risk tumors, contrary to the World statistics that point to lower adjusted incidence and mortality rates for this group (1), arguing about possible genetic mutations in people who immigrated to the Western countries, especially to Brazil (33). The great miscegenation observed in the Brazilian population among Caucasians, Afro-descendants, Eastern descendants, and Native Brazilian Indians could explain this behavior, however this is only an assumption that could be unveiled by genetic studies. According to the Epstein's criteria, our cohort showed 16.3% (41 patients) of clinically indolent prostate cancer cases, considerably lower than the 24% to 25% reported in literature (34).

Age-oriented strategies for screening are also conflictual. Our study started to screening patients over 40 years old, with no age limit. PCa incidence in patients aged 40-49 years was extremely low with only 10 diagnoses out of 3.280 (0.3%), questioning the reliability of screening for this age group. On the other hand, PPV of this strategy in this age group was 38.5%, higher than the previously reported by Faria et al. in a similar Brazilian population (26.7%) (29). In addition, 50% of these patients were diagnosed with high-risk disease, which is known to have an impact on survival, increasing the importance of discussing specific screening strategies for this group, instead of discuss if we have to do or not do PSA-screening (35). Median age of 54 years for screened patients can also be considered low, since PCa incidence is higher in the population aged 55-69 years (1, 8, 29).

A recent study from Mori et al. (36) made a cross-sectional analysis involving more than 17.000 men, with 18.7% of volunteers aged over 70 years. They found a higher prevalence of PCa, higher PSA levels, and more aggressive or metastatic disease among elderly men. In our series, men ≥70 years presented 34.6% of the PCa cases, and almost 44% of the high-risk diseases. Considering the increase in life expectancy and the non-inclusion of this age group in screening policies, it is crucial to inform this evidence to elderly men requesting pertinent guidance.

Regarding metastatic disease, incidence of 4.3% was lower when compared to 6% in the United States data (20), and even when compared to other previously published Brazilian data, which demonstrated higher incidences ranging from 16.5% to 26.5% of patients in clinical stage IV in a non-screened population (18, 19).

After the USPSTF recommendation against PSA-screening, incidence rates declined in the United States followed by increasing trends in metastatic disease since 2012 (35, 37–40). Subsequently, USPSTF changed recommendation level from “D” to “C”, assuming that there is a potential benefit in mortality rates in PSA-screening among patients aged 55-69 years old (40). For further analysis of the impact in PCa incidence after the previous USPSTF recommendations in 2012, Jemal et al. examined the trends of more than 2 million men up to 2016. Regional disease increased by 11% from 2012-2016, and metastatic disease increased from 2010-2016 by 5.0% per year. Reasons not fully understood for this burden impacted particularly black men in the US (41). In the same way, several studies proved an increase in detection of Gleason scores 7-10 in diagnosed patients after 2012 (42, 43).

The retrospective nature of this study carried out in a single center and for a short period of time (1 year) are limiting factors in this analysis. On the other hand, this screening period can considerably reduce the effects of contamination in samples among screened and non-screened patients, which was an important concern identified in the PLCO, with 44% of contamination in the control group (5). Current guidelines emphasize the evidences of mpMRI prior to the biopsy, when available. They showed an improvement in the PCa diagnosis classified as ISUP ≥2 in both biopsy-naïve patients or in those with prior negative biopsy (44). In 2013, this was not a standard practice and mpMRI was not performed previously to the diagnosis. In the same way, despite of current evidence, patients in this study had no access to fusion biopsy, particularly in the public health setting. Another limiting factor was the failure to perform DRE in the population with PSA>10.0ng/dL and the failure to perform fPSA test for the entire cohort, which makes it difficult to compare these data with the literature. DRE performed by non-specialists decreases sensitivity and specificity of this approach. However, this is more in line with the primary care setting, where most men in the public health system will look for medical assistance. Based on our data, we cannot be aware about optimal testing intervals. Despite that, we could describe the profile of a large group of men who volunteered for screening. Related to the calculated PPV, it is noted that the prevalence of disease impacts this value. PPV is not intrinsic to a test and this is another limitation of our analysis.

## CONCLUSIONS

Our analysis described the results of a cancer prevention program at a Brazilian reference cancer center performed by non-urological medical staff. This PSA-screening strategy found a comparable incidence rate of PCa compared to other national or international studies. However, we noticed a higher incidence of high-risk disease in this population, particularly regarding black or yellow men.

In front of a huge miscegenated Brazilian population and considering the big proportion of high-risk PCa, even in young men diagnosed with the disease (age <50 years), it is imperative to inform patients and health providers about these data particularities in Brazil. More comprehensive studies are needed to support decision, especially in the public health scope.

## References

[1] 1. [No Authors]. International Agency for Research on Cancer (IARC). Globocan 2018: Global Cancer Observatory-GCO. [Internet]. Availate at. <http://gco.iarc.fr>. Acccess in Jan 12, 2020

[2] 2. Estimativa 2020. Estimativas para o ano 2020 de número de casos novos de câncer. [Internet]. Availate at. <https://www.inca.gov.br/estimativa/estado-capital/brasil>

[3] 3. Etzioni R, Gulati R, Tsodikov A, Wever EM, Penson DF, Heijnsdijk EA, et at. The prostate cancer conundrum revisited: treatment changes and prostate cancer mortality declines. Cancer. 2012; 118:5955-63. 10.1002/cncr.27594 PMC3424303 22605665

[4] 4. Hoffman RM. Clinical practice. Screening for prostate cancer. N Engl J Med. 2011; 365:2013-9. 10.1056/NEJMcp1103642 22029754

[5] 5. Andriole GL, Crawford ED, Grubb RL 3rd, Buys SS, Chia D, Church TR, et al. Project Team. Mortality results from a randomized prostate-cancer screening trial. N Engl J Med. 2009; 360:1310-9. Erratum in: N Engl J Med. 2009; 360:17971797. 10.1056/NEJMoa0810696 PMC2944770 19297565

[6] 6. Ilic D, O’Connor D, Green S, Wilt T. Screening for prostate cancer. Cochrane Database Syst Rev. 2006; 3:CD004720. Cochrane Database Syst Rev. 2013;1:CD004720CD004720. 10.1002/14651858.CD004720.pub2 16856057

[7] 7. Schröder FH, Hugosson J, Roobol MJ, Tammela TL, Ciatto S, Nelen V, et at. Screening and prostate-cancer mortality in a randomized European study. N Engl J Med. 2009; 360:1320-8. 10.1056/NEJMoa0810084 19297566

[8] 8. Schröder FH, Hugosson J, Roobol MJ, Tammela TL, Zappa M, Nelen V, et at. Screening and prostate cancer mortality: results of the European Randomised Study of Screening for Prostate Cancer (ERSPC) at 13 years of follow-up. Lancet. 2014; 384:2027-35. 10.1016/S0140-6736(14)60525-0 PMC4427906 25108889

[9] 9. Hugosson J, Carlsson S, Aus G, Bergdahl S, Khatami A, Lodding P, et at. Mortality results from the Göteborg randomised population-based prostate-cancer screening trial. Lancet Oncol. 2010; 11:725-32. 10.1016/S1470-2045(10)70146-7 PMC4089887 20598634

[10] 10. Wilt TJ, Brawer MK, Jones KM, Barry MJ, Aronson WJ, Fox S, et at. Radical prostatectomy versus observation for localized prostate cancer. N Engl J Med. 2012; 367:203-13. Erratum in: N Engl J Med. 2012; 367:582582. 10.1056/NEJMoa1113162 PMC3429335 22808955

[11] 11. Wilt TJ, Jones KM, Barry MJ, Andriole GL, Culkin D, Wheeler T, et at. Follow-up of Prostatectomy versus Observation for Early Prostate Cancer. N Engl J Med. 2017; 377:132-42. 10.1056/NEJMoa1615869 28700844

[12] 12. Hamdy FC, Donovan JL, Lane JA, Mason M, Metcalfe C, Holding P, et at. 10-Year Outcomes after Monitoring, Surgery, or Radiotherapy for Localized Prostate Cancer. N Engl J Med. 2016; 375:1415-24. 10.1056/NEJMoa1606220 27626136

[13] 13. Donovan JL, Hamdy FC, Lane JA, Mason M, Metcalfe C, Walsh E, et at. Patient-Reported Outcomes after Monitoring, Surgery, or Radiotherapy for Prostate Cancer. N Engl J Med. 2016; 375:1425-37. 10.1056/NEJMoa1606221 PMC5134995 27626365

[14] 14. Rawla P. Epidemiology of Prostate Cancer. World J Oncol. 2019; 10:63-89. 10.14740/wjon1191 PMC6497009 31068988

[15] 15. Kheirandish P, Chinegwundoh F. Ethnic differences in prostate cancer. Br J Cancer. 2011; 105:481-5. 10.1038/bjc.2011.273 PMC3170971 21829203

[16] 16. Center MM, Jemal A, Lortet-Tieulent J, Ward E, Ferlay J, Brawley O, et at. International variation in prostate cancer incidence and mortality rates. Eur Urol. 2012; 61:1079-92. 10.1016/j.eururo.2012.02.054 22424666

[17] 17. Cooperberg MR, Cowan J, Broering JM, Carroll PR. High-risk prostate cancer in the United States, 1990-2007. World J Urol. 2008; 26:211-8. 10.1007/s00345-008-0250-7 PMC2948572 18369637

[18] 18. [No Authors]. Sobrevida de Pacientes com Câncer no estado de São Paulo: seis anos de seguimento pelo registro hospitalar de câncer. Cadernos Fosp Vol.5. Secretaria de Estado da saúde de São Paulo, Fundação Oncocentro de São Paulo. [Internet]. Available at. <http://www.fosp.saude.sp.gov.br:443/epidemiologia/docs/sobevida.pdf>

[19] 19. Carneseca EC, Mauad EC, de Araujo MA, Dalbó RM, Longatto Filho A, Vazquez Vde L. The Hospital de Câncer de Barretos Registry: an analysis of cancer survival at a single institution in Brazil over a 10-year period. BMC Res Notes. 2013; 6:141. 10.1186/1756-0500-6-141 PMC3637553 23574710

[20] 20. [No Authors]. Cancer Stat Facts: Prostate Cancer. National Cancer Institute. [Internet]. Available at. <https://seer.cancer.gov/statfacts/html/prost.html>

[21] 21. Epstein JI, Egevad L, Amin MB, Delahunt B, Srigley JR, Humphrey PA. The 2014 International Society of Urological Pathology (ISUP) Consensus Conference on Gleason Grading of Prostatic Carcinoma: Definition of Grading Patterns and Proposal for a New Grading System. Am J Surg Pathol. 2016; 40:244-52. 10.1097/PAS.0000000000000530 26492179

[22] 22. Amin MB, Edge S, Greene F, Byrd DR, Brookland RK, Washington MK, AJCC Cancer Staging Manual. New York, Springer. 2017.

[23] 23. Moyer VA; U.S. Preventive Services Task Force. Screening for prostate cancer: U.S. Preventive Services Task Force recommendation statement. Ann Intern Med. 2012; 157:120-34. 10.7326/0003-4819-157-2-201207170-00459 22801674

[24] 24. Catalona WJ. Prostate Cancer Screening. Med Clin North Am. 2018; 102:199-214. 10.1016/j.mcna.2017.11.001 PMC5935113 29406053

[25] 25. Grenabo Bergdahl A, Holmberg E, Moss S, Hugosson J. Incidence of prostate cancer after termination of screening in a population-based randomised screening trial. Eur Urol. 2013; 64:703-9. Erratum in: Eur Urol. 2015; 68:e46. 10.1016/j.eururo.2013.05.024 23721957

[26] 26. Godtman RA, Carlsson S, Holmberg E, Stranne J, Hugosson J. The Effect of Start and Stop Age at Screening on the Risk of Being Diagnosed with Prostate Cancer. J Urol. 2016; 195:1390-6. 10.1016/j.juro.2015.11.062 PMC4948858 26678954

[27] 27. Tourinho-Barbosa RR, Pompeo AC, Glina S. Prostate cancer in Brazil and Latin America: epidemiology and screening. Int Braz J Urol. 2016; 42:1081-90. 10.1590/S1677-5538.IBJU.2015.0690 PMC5117963 27622278

[28] 28. [No Authors]. 2020 IBGE - Instituto Brasileiro de Geografia e Estatística. [Internet]. Available at. <https://censo2010.ibge.gov.br/index.php>. Accessed in Mar 9, 2020.

[29] 29. Faria EF, Carvalhal GF, Vieira RA, Silva TB, Mauad EC, Carvalho AL. Program for prostate cancer screening using a mobile unit: results from Brazil. Urology. 2010; 76:1052-7. 10.1016/j.urology.2010.02.044 20472277

[30] 30. Catalona WJ, Richie JP, Ahmann FR, Hudson MA, Scardino PT, Flanigan RC, et at. Comparison of Digital Rectal Examination and Serum Prostate Specific Antigen in the Early Detection of Prostate Cancer: Results of a Multicenter Clinical Trial of 6,630 Men. J Urol. 2017; 197:S200-S207. 10.1016/j.juro.2016.10.073 28012755

[31] 31. Crawford ED, Leewansangtong S, Goktas S, Holthaus K, Baier M. Efficiency of prostate-specific antigen and digital rectal examination in screening, using 4.0 ng/ml and age-specific reference range as a cutoff for abnormal values. Prostate. 1999; 38:296-302. 10.1002/(sici)1097-0045(19990301)38:4<296::aid-pros5>3.0.co;2-p 10075009

[32] 32. Chu LW, Ritchey J, Devesa SS, Quraishi SM, Zhang H, Hsing AW. Prostate cancer incidence rates in Africa. Prostate Cancer. 2011;2011:947870. 10.1155/2011/947870 PMC3200287 22111004

[33] 33. Zhu Y, Wang HK, Qu YY, Ye DW. Prostate cancer in East Asia: evolving trend over the last decade. Asian J Androl. 2015; 17:48-57. 10.4103/1008-682X.132780 PMC4291877 25080928

[34] 34. Singh H, Canto EI, Shariat SF, Kadmon D, Miles BJ, Wheeler TM, et at. Improved detection of clinically significant, curable prostate cancer with systematic 12-core biopsy. J Urol. 2004; 171:1089-92. 10.1097/01.ju.0000112763.74119.d4 14767277

[35] 35. Eapen RS, Herlemann A, Washington SL 3rd, Cooperberg MR. Impact of the United States Preventive Services Task Force ‘D’ recommendation on prostate cancer screening and staging. Curr Opin Urol. 2017; 27:205-9. 10.1097/MOU.0000000000000383 28221220

[36] 36. Mori RR, Faria EF, Mauad EC, Rodrigues AA Jr, Dos Reis RB. Prostate cancer screening among elderly men in Brazil: should we diagnose or not? Int Braz J Urol. 2020; 46:34-41. 10.1590/S1677-5538.IBJU.2019.0022 PMC6968892 31851456

[37] 37. Fleshner K, Carlsson SV, Roobol MJ. The effect of the USPSTF PSA screening recommendation on prostate cancer incidence patterns in the USA. Nat Rev Urol. 2017; 14:26-37. 10.1038/nrurol.2016.251 PMC5341610 27995937

[38] 38. Li J, Siegel DA, King JB. Stage-specific incidence rates and trends of prostate cancer by age, race, and ethnicity, United States, 2004-2014. Ann Epidemiol. 2018; 28:328-30. 10.1016/j.annepidem.2018.03.001 PMC6080305 29678312

[39] 39. Kearns JT, Holt SK, Wright JL, Lin DW, Lange PH, Gore JL. PSA screening, prostate biopsy, and treatment of prostate cancer in the years surrounding the USPSTF recommendation against prostate cancer screening. Cancer. 2018; 124:2733-2739. 10.1002/cncr.31337 29781117

[40] 40. US Preventive Services Task Force, Grossman DC, Curry SJ, Owens DK, Bibbins-Domingo K, Caughey AB, et at. Screening for Prostate Cancer: US Preventive Services Task Force Recommendation Statement. JAMA. 2018; 319:1901-1913. Erratum in: JAMA. 2018; 319:2443. 10.1001/jama.2018.3710 29801017

[41] 41. Jemal A, Culp MB, Ma J, Islami F, Fedewa SA. Prostate Cancer Incidence 5 Years After US Preventive Services Task Force Recommendations Against Screening. J Natl Cancer Inst. 2020, Epub ahead of print. 10.1093/jnci/djaa068 PMC7781461 32432713

[42] 42. Bhindi B, Mamdani M, Kulkarni GS, Finelli A, Hamilton RJ, Trachtenberg J, et at. Impact of the U.S. Preventive Services Task Force recommendations against prostate specific antigen screening on prostate biopsy and cancer detection rates. J Urol. 2015; 193:1519-24. 10.1016/j.juro.2014.11.096 25481037

[43] 43. Gejerman G, Ciccone P, Goldstein M, Lanteri V, Schlecker B, Sanzone J, et at. US Preventive Services Task Force prostate-specific antigen screening guidelines result in higher Gleason score diagnoses. Investig Clin Urol. 2017; 58:423-428. Erratum in: Investig Clin Urol. 2018; 59:283. 10.4111/icu.2017.58.6.423 PMC5671961 29124241

[44] 44. Mottet N, Cornford P, van den Bergh RCN, Briers E, De Santis M, Fanti S, et al. EAU-EANM-ESTRO-ESUR-SIOG Guidelines on Prostate Cancer. Edn. presented at the EAU Annual Congress Amsterdam 2020. EAU Guidelines Office. [Internet]. Available at. <https://uroweb.org/wp-content/uploads/2020-EAU-EAUNM-ESTRO-ESUR-SIOG-Prostate-Cancerr-Guidelines-Search-Strategy.pdf>.

